# Microbial Safety of Dairy Manure Fertilizer Application in Raspberry Production

**DOI:** 10.3389/fmicb.2019.02276

**Published:** 2019-10-02

**Authors:** Lina Sheng, Xiaoye Shen, Chris Benedict, Yuan Su, Hsieh-Chin Tsai, Elizabeth Schacht, Chad E. Kruger, Margaret Drennan, Mei-Jun Zhu

**Affiliations:** ^1^School of Food Science, Washington State University, Pullman, WA, United States; ^2^Whatcom County Extension, Washington State University, Bellingham, WA, United States; ^3^Center for Sustaining Agriculture and Natural Resources, Washington State University, Pullman, WA, United States; ^4^Natural Resources Assessment Section, Washington State Department of Agriculture, Olympia, WA, United States

**Keywords:** raspberry, fertilizer, soil, foliar, indicator microorganisms, pathogenic microorganisms

## Abstract

Dairy manure, a by-product in the dairy industry, is also a potential source of nutrients for crops. However, improper application of biological soil amendments of animal origin can be a source of contamination with enteric foodborne pathogens. A 2-year field study was conducted to evaluate impacts of dairy manure fertilizer application on the microbial safety of red raspberry (*Rubus idaeus* L) production. Fertilizers, including a standard synthetic fertilizer (CON), straight lagoon raw manure (SL), anaerobically digested liquid effluent (DLE), compost (COM) and dairy manure-derived refined fertilizers including ammonium sulfate (AS) and phosphorous solid (PS), were randomly applied in quadruplicate to raspberry plots. Soil, fertilizer, foliar, and raspberry fruit samples were collected during the cropping season for the quantification of indicator microorganisms (total coliform and generic *Escherichia coli*) and detection of important foodborne pathogens (Shiga toxin-producing *E. coli* (STEC), *Salmonella*, and *Listeria monocytogenes*). Counts of total coliforms in soil were stable over the 2017 cropping season and were not impacted by fertilizer application. In 2018, total coliforms increased with season and soils treated with COM had a significantly higher coliform number than those treated with CON. Both total coliform and generic *E. coli* in raspberry fruit samples were below the detectable level (3 most probable number/g) regardless of fertilizer types. In both years, no STEC or *L. monocytogenes* was detected from any of the collected samples regardless of fertilizer treatments. However, *Salmonella* were detected in some of the fertilizers, including PS (2017), DLE (2018), and SL (2018), which were transferred to soil samples taken directly after application of these fertilizers. *Salmonella* were not detected in soil samples 2 or 4 months post fertilizer application, foliar, or raspberry fruit samples regardless of fertilizer applications. In summary, one-time application of raw dairy manure or dairy manure-derived fertilizers more than 4 months prior to harvest has no major impact on food safety of red raspberry (6 ft. tall) production in Lynden sandy loam under good agricultural practices.

## Introduction

Dairy-derived fertilizers are high in nutrients such as nitrogen and phosphate and can increase organic matter content in soil and subsequently improve soil fertility and physical properties such as aggregation, resistance to water or wind erosion, and water-holding capacity (Carter and Stewart, 1995; Rechcigl, 1995; Zebarth et al., 1999). Application of dairy manure-derived fertilizers in a raspberry field can enhance plant root growth and nutrient acquisition (Forge et al., 2014). However, raw manure potentially carries different pathogens such as Shiga toxin-producing *Escherichia coli* (STEC), *Salmonella* spp., and *Listeria* spp. (Hutchison et al., 2005), which are three major pathogens frequently involved in fresh produce outbreaks. A high level of 2.6 × 10^8^ CFU/g of *E. coli* O157:H7 was detected in fresh cattle manure (Hutchison et al., 2004). The National Organic Program (7 CFR §205.203) requires that raw animal manure must be composted before application to crops for human consumption or incorporated into the soil more than 120 days prior to harvest for produce whose edible portion has direct contact with soil or 90 days prior to harvest for crops that have no direct contact with soil (CFR, 2019). The Produce Safety Rule under Food Safety Modernization Act (FSMA) also emphasizes that the application of raw manure must not contact produce during application and the potential for contact with produce after application should be minimized (FDA, 2019b). Federal Regulations (21CFR122.55) further specified the microbial standards for biological soil amendments of animal origin including less than 0.3 most probable number (MPN) per gram or milliliter of analytical portion for *E. coli* O157:H7, less than 3 MPN per 4 g or ml of total solids for *Salmonella* spp., and less than 1 colony-forming unit per 5 g or ml of analytical portion for *L. monocytogenes* (FDA, 2018b).

Therefore, prior to field application, dairy manure is commonly subjected to different treatments such as composing (FDA, 2018a) and anaerobic digestion (Wilkie, 2005) to reduce pathogen level and meet the above microbiological standards. The prediction of pathogen reduction during manure handling and field application is difficult due to the lack of lab or pilot field studies and various influencing factors such as temperature, duration, pH, and moisture (Sobsey et al., 2006). Previous studies have also reported the persistence and survival of pathogens during manure treatments such as storage, piling, composting, and anaerobic digestion (Grewal et al., 2007; Resende et al., 2014), posing risk to the safety of fresh produce production systems. During manure application, the potential incorporated pathogens can further be transferred to soil, which also serves as a reservoir of foodborne pathogens (Natvig et al., 2002; Islam et al., 2004b, 2005; Mootian et al., 2009), or even directly contaminate the fresh produce through aerosols under harsh weather such as strong winds (Baertsch et al., 2007). It was reported that *E. coli* O157:H7 (Islam et al., 2004a) and *S.* Typhimurium (Islam et al., 2004b) persisted for more than 200 days in soil amended with composts. Survival of pathogens in soil is further influenced by soil type (Jechalke et al., 2019). Pathogens in soil can be transferred to fresh produce grown in contaminated soil (Hirneisen et al., 2012; Eissenberger et al., 2018; Jechalke et al., 2019). Therefore, raw manure and manure-derived fertilizers can become a contamination source to introduce pathogenic bacteria into fresh produce, causing serious foodborne outbreaks. Dairy manure was identified as a potential contamination source for the 2018 multistate outbreak of *E. coli* O157:H7 linked to romaine lettuce (210 cases and 5 deaths) (CDC, 2018a). A multistate *E. coli* O157:H7 outbreak associated with spinach due to dairy manure infected over 200 people and caused three deaths in 26 states (Grant et al., 2008).

Berries are grown in open environments and subjected to pathogenic microorganism contamination during production (Macori et al., 2018; Tefera et al., 2018). Raspberry and raspberry products have been involved in outbreaks and recalls associated with human norovirus (Sarvikivi et al., 2012; CFIA, 2017; FDA, 2019a,c) and *Cyclospora cayetanensis* (Herwaldt et al., 1997; Ho et al., 2002). Fresh blueberries were involved in a *Salmonella* Newport outbreak in Minnesota, 2010 (Miller et al., 2013). In addition, raspberries are usually not subjected to any antimicrobial treatment before packing or being consumed as fresh berry due to the delicate and complicated structure. Any contamination in a raspberry field, including improperly treated compost or raw manure, irrigation water, soil, and wild or domestic animals (Beuchat, 2002), can pose a threat to food safety and human health. Foodborne pathogens *L. monocytogenes*, *Salmonella* spp., and STEC particularly *E. coli* O157:H7 have been frequently involved in various fresh produce outbreaks (Grant et al., 2008; CDC, 2011, 2012; FDA, 2014) and are of the greatest public health concerns. However, to our knowledge, no document exists regarding the potential risk of the above three foodborne pathogens during raspberry production. Once pathogens are introduced to raspberries, *Salmonella* and *E. coli* O157:H7 were able to survive on raspberry at 4°C for at least 10 days (Xu and Wu, 2016). Therefore, it is of great importance for the raspberry industry to evaluate the microbiological safety of the current production. The objective of this study was to assess the microbial risk of dairy manure fertilizer application in a red raspberry cropping system.

## Materials and Methods

### Field Experimental Design

The field experiment was conducted in a 4.79-acre commercial red raspberry (*Rubus idaeus* L) field located in Whatcom County, Washington State in 2017 and 2018. The red raspberries were grown on Lynden sandy loam, a common soil type for red raspberries grown in this area, which is a deep and well-drained soil. The field was planted to the floricane fruiting variety “Meeker,” the most common red raspberry variety for the processing industry. This commercial raspberry farm had been managed similarly since its planting in 2011. Floricanes are removed annually after fruiting in the fall or winter and trained to a trellis. Bare soil (Lynden sandy loam) is maintained below plants for the application of fertilizers. Individual plot (22.86 m × 3.05 m) contains one row of raspberry plant. There is a buffer row between treatments (6.10 m buffer between treatment rows) to isolate plots and prevent cross-contamination.

Treatments included a standard synthetic granular fertilizer (CON) acquired from a local fertilizer dealer, aerobically composted diary manure (COM) purchased from a local compost company that uses local dairy manure as a feedstock, raw manure straight lagoon (SL) and anaerobically digested diary manure products including digested liquid effluent (DLE), phosphorous solids (PS) and ammonium sulfate (AS) that were collected from one local dairy farm. Fertilizers extracted from anaerobic digestion were previously summarized in Benedict et al. (2018). Briefly, after anaerobic digestion of SL and subsequent physical separation of low-nutrient fibrous solids, PS is extracted from the remaining effluent using centrifuge or dissolved air flotation systems. AS is derived from the manure products post-PS extraction through ammonia stripping process. DLE is the liquid portion of manure that had passed through the anaerobic digestion. Physiochemical properties including moisture, pH, and nutritional information of each fertilizer are summarized in Table 1. CON was applied using standard side-discharge fertilizer spreader and the product was placed into a 0.06 m band beneath the raspberry plants. AS was applied by CO_2_ backpack sprayer. COM and PS were hand-applied. DLE and SL were applied using custom in-row manure spreader. The fertilizers were applied based on agronomic rates (nitrogen (N)/acre for AS, COM; phosphorus (P) for DLE, PS, SL; nitrogen-phosphorus-potassium (NPK) for CON). We were shooting to deliver the same total NPK based on what the growers considered “standard” for their cropping systems. All fertilizers were applied once per season following good agricultural practices. Plots were setup in a completely randomized design with four replications per fertilizer treatment. The plots were irrigated with well water using two buried drip lines.

**Table 1:** Physiochemical properties of tested fertilizers.

Fertilizer	Moisture (%)	Nitrogen		Phosphorus (g P_2_O_5_/kg)	Potassium (g K_2_O/kg)	pH
		NH_4_ (g/kg)	Total N (g/kg)			
CON	/	210	210	440	500	/
AS	100.0	52.4	50.3	0.002	0.01	1.77
COM	69.5	1.8	21.5	12.8	18.5	8.02
DLE	96.5	1.5	2.5	0.4	1.8	/
PS	70.3	4.7	32.4	31.6	11.3	8.62
SL	95.6	0.9	1.8	0.3	2.8	/

*Values are averaged from 12 samples/fertilizer over the 2-year study (6 samples/year, *n* = 12). /: not measured; CON: standard fertilization; AS: ammonium sulfate; COM: compost; DLE: digested liquid effluent; PS: phosphorous solid; SL: straight lagoon*.

### Sampling Plan

**Figure 1:**
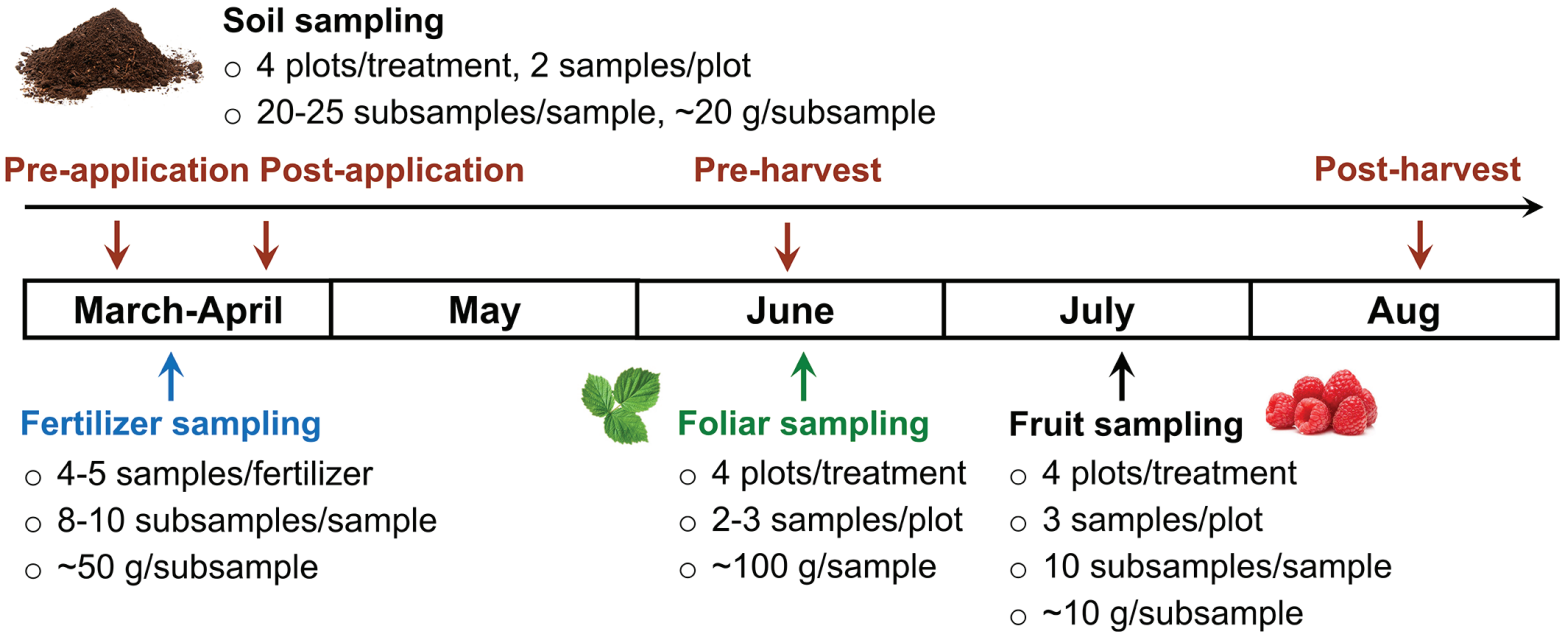
Microbiological sample collection plan in 2017 and 2018 cropping seasons.

Sampling plan is outlined in Figure 1. Fertilizers were sampled before application in late March; five samples were collected per fertilizer in 2017 and four samples per fertilizer in 2018. CON was sampled from 5-gallon buckets with 8–10 subsamples (~50 g/subsample) per sample. COM and PS were piled (~3 m × 3 m, 1.2 m height) and collected in plastic bags from different locations of the pile with 8–10 subsamples (~50 g/subsample) taken from three depths per location. For liquid or slurry fertilizers (AS, DLE, SL), samples were collected in 1-L polypropylene bottles from different location of the holding tank. Soil samples were collected before fertilizer application, right after fertilizer application, and 2 months post-application (1 month pre-harvest), and 1 month post-harvest (Figure 1). Two samples were collected per plot with 20 subsamples per sample (~500 g/sample) using a soil core sampler (2.5 cm diameter) from a depth of 0–5 cm (EPA, 2014). Foliar samples (~100 g/sample, ~50 leaves) were collected in brown paper bags in triplicate per plot for 2017 study and in duplicate per plot for 2018 study before fruit harvest. Fully expanded leaves devoid of disease or any other damage were picked all over the canopy (~1 m off the ground) including primocane and floricane leaves. Raspberry fruits of commercial maturity were sampled on the day prior to the beginning of commercial harvest. Three samples (~100 g/sample, ~30 raspberries) were randomly collected from 10 different areas or bushes per plot. The above samples were transported on ice in an insulated cooler to Food Microbiology laboratory and analyzed within 24 h.

### Quantification of Indicator Microorganisms

Bacteria of enteric origin such as generic *E. coli* and total coliforms are commonly used as indicator microorganisms to assess the potential presence of fecal contamination in water and compost (EPA, 2012; Danyluk et al., 2013). However, coliforms are reported as a poor indicator for fresh fruits and vegetables because they are part of the normal microbiota (Danyluk et al., 2013). Suitability of total coliforms and generic *E. coli* as fecal indicator microorganisms were assessed in the raspberry production system. Total coliforms and generic *E. coli* were enumerated by both direct plating and 3-tube MPN (FDA, 2002). Briefly, 25 g of representative sample was homogenized in 225 ml of buffered peptone water [BPW, Becton, Dickinson and Company (BD), Sparks, MD]. The resulting bacterial suspension was serially diluted and spread onto duplicate CHROMagar ECC (CHROMagar, Paris, FR) plates and incubated at 30 and 44.5°C for 24 h for direct enumeration of total coliform and generic *E. coli,* respectively. The limit of detection (LOD) for the plating method is 10 CFU/g. For MPN estimation, 1 ml of the above bacterial suspension was transferred to three tubes containing 9 ml of lauryl tryptose broth [LST, Hardy Diagnostics (HD), Santa Maria, CA] with a Durham tube. LST tubes were incubated at 35°C for 24–48 h for gas and turbidity. Gas-positive turbid tubes were further transferred with a sterile loop to brilliant green lactose bile broth (BGLB, HD) and *E. coli* broth with 4-methylumbelliferyl-β-D-glucuronide (EC + MUG, HD) for the confirmation of total coliform and generic *E. coli,* respectively. BGLB tubes were incubated at 35°C for 24–48 h. EC + MUG tubes were incubated at 44.5°C for 24 h. Turbid BGLB tubes with gas production were considered coliform positive. Turbid EC + MUG tubes with gas production and fluorescence under a long-wave UV lamp were considered generic *E. coli* positive. MPN results were interpreted according to Bacteriological Analytical Manual (BAM) (FDA, 2010). The LOD for MPN method is 3 MPN/g.

### Detection of Shiga Toxin-Producing *E. coli*

STEC were detected using both standard plating method and multiplex PCR (Feng et al., 2011). Briefly, 25 g of representative sample was homogenized in 225 ml of modified BPW with 0.1% (w/v) pyruvate (Amresco, Solon, OH), incubated at 35°C for 5 h, then supplemented with selective reagents to final concentrations of 10 mg/L acriflavin (TCI America, Portland, OR), 10 mg/L cefsulodin (Sigma-Aldrich, St. Louis, MO), and 8 mg/L vancomycin (Sigma-Aldrich), mixed and incubated at 42°C for additional 18–24 h. The overnight culture was also serially diluted and spread onto CHROMagar STEC (CHROMagar) plates for the isolation of STEC. For multiplex PCR detection, the overnight enrichment was used to extract DNA and subjected to multiplex PCR with fluorescent probes targeting for *stx1*, *stx2*, and *uidA* encoding for the β-D-glucuronidase enzyme for the rapid detection of STEC (Feng et al., 2011). The LOD for STEC is 1 CFU/g.

### Detection of *Salmonella*

*Salmonella* spp. were detected by standard culturing method and confirmed by latex agglutination and PCR (FDA, 2011). Briefly, 25 g of representative samples was homogenized in 225 ml of BPW and incubated at 35°C for 24 h, which was sub-cultured in Rappaport-Vassiliadis (RV, HD) and tetrathionate (TT, BD) broth for selective enrichment at 42 and 35°C for 24 h, respectively. The resulting enrichment culture was streaked onto xylose lysine desoxycholate (XLD, HD), bismuth sulfite (BS, HD), hektoen enteric (HE, HD), and CHROMagar *Salmonella* (CHROMagar) plates and incubated at 35°C for 24–48 h. The presumptive positive colonies were confirmed with *Salmonella* Latex Test (Oxoid Ltd., Hants, UK) and PCR targeting *Salmonella* invasion gene *invA* (Rahn et al., 1992; Malorny et al., 2003). A sample is positive when *Salmonella* is found in any combination of enrichment media and plates. *Salmonella* positive samples were further subjected to enumeration using three-tube MPN method following published method (Fravalo et al., 2003). The LOD for MPN enumeration is 3 MPN/g.

### Detection of *L. monocytogenes*

*L. monocytogenes* in the samples was detected by standard plating technique. Briefly, 25 g of fertilizer, soil, foliage, or fruit sample was homogenized in 225 ml of buffered *Listeria* enrichment broth (BLEB, BD), non-selectively enriched for 4 h at 30°C, followed by additional 24–48 h of selective enrichment with 10 mg/L acriflavin, 50 mg/l cycloheximide (Amresco), and 40 mg/L nalidixic acid (Sigma-Aldrich). The enrichment culture was streaked onto modified Oxford agar (MOX, BD) and CHROMagar *Listeria* (CHROMagar) plates. MOX and CHROMagar *Listeria* plates were incubated at 35 and 37°C, respectively, for 24–48 h. The presumptive positive colonies were confirmed by PCR targeting invasion-associated secreted endopeptidase (*iap*) gene (FDA, 2017).

### Statistical Analysis

Quantification data were analyzed by GLM from Statistical Analysis Systems (SAS, Cary, NC). Mean values were compared by least significant difference (LSD) multiple-comparison test. Values of *p* less than 0.05 were considered significant. Results were reported as mean ± SEM (standard error of mean).

## Results

### Quantification of Indicator Microorganisms

**Figure 2:**
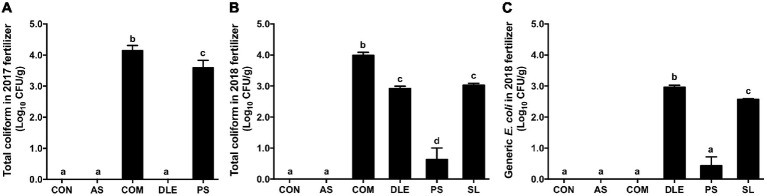
Abundance of total coliform and generic *E. coli* in fertilizers used in raspberry field 2017–2018. **(A)** Total coliform counts of fertilizers in 2017; **(B)** total coliform enumeration of fertilizers in 2018; **(C)** Generic *E. coli* enumeration of fertilizers in 2018. Mean ± SEM, five samples were collected per fertilizer in 2017 and four samples per fertilizer in 2018; 25 g per sample was tested. Histogram bars without common letter differ significantly (*p* < 0.05). CON: standard fertilization; AS: ammonium sulfate; COM: compost, DLE: digested liquid effluent; PS: phosphorous solid; SL: straight lagoon.

In 2017, 4.14 ± 0.08 and 3.60 ± 0.10 Log_10_ CFU/g total coliform were detected in COM and PS, respectively, while those in CON, AS, and DLE were under detection limit (3 MPN/g) (Figure 2A). Consistent with 2017, total coliform in CON and AS were below the detection limit and that in COM was 3.99 ± 0.10 Log_10_ CFU/g (Figure 2B). While indicator microorganisms in DLE, PS, and SL were 2.92 ± 0.07, 0.63 ± 0.37, and 3.03 ± 0.06 Log_10_ CFU/g, respectively, for total coliform, and 2.97 ± 0.04, 0.43 ± 0.28, and 2.57 ± 0.02 Log_10_ CFU/g, respectively for generic *E. coli* (Figures 2B,C).

**Figure 3:**
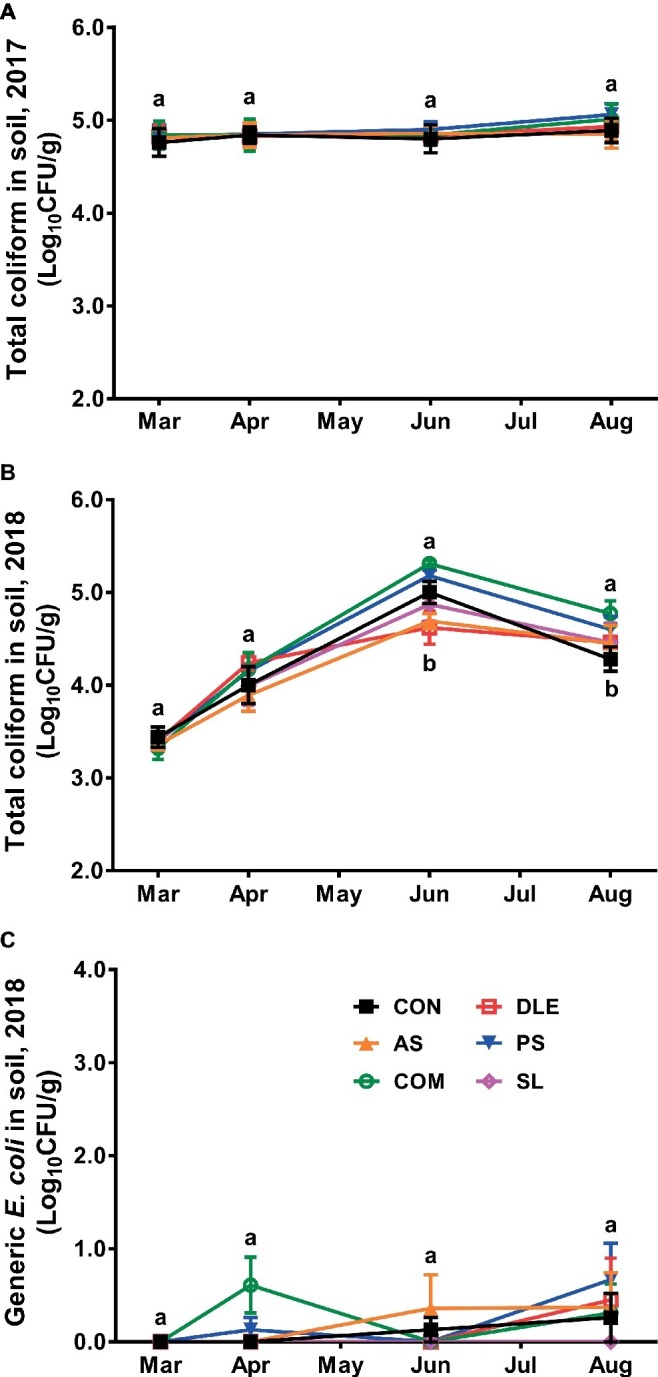
Enumeration of total coliform and generic *E. coli* in soil samples of raspberry field in both 2017 and 2018 cropping seasons. **(A)** Total coliform 2017; **(B)** total coliform 2018; **(C)** generic *E. coli* 2018. Mean ± SEM, eight samples were collected per treatment and 25 g per sample was tested. Mean values at each sampling point without common letter differ significantly (*p* < 0.05). CON: standard fertilization; AS: ammonium sulfate; COM: compost, DLE: digested liquid effluent; PS: phosphorous solid; SL: straight lagoon.

Total coliform in soil samples before fertilizer application in 2017 study was 4.84 ± 0.03 Log_10_ CFU/g, and it remained at similar levels throughout the cropping system regardless of fertilizer treatment (Figure 3A). In 2018, however, total coliform in soil samples before fertilizer application was 3.38 ± 0.04 Log_10_ CFU/g, which increased by 0.4–0.6 Log_10_ CFU/g right after fertilizer application independent of the treatment (Figure 3B). Total coliform was further increased in soil samples during the subsequent 2 months and the count increased most in COM while least in DLE (Figure 3B). The total coliform decreased in general over the season; its counts in soil with COM fertilizer were significantly (*p* < 0.05) higher than those in CON group (Figure 3B). Generic *E. coli* counts remained less than 1.0 Log_10_ CFU/g throughout the season (Figure 3C).

**Figure 4:**
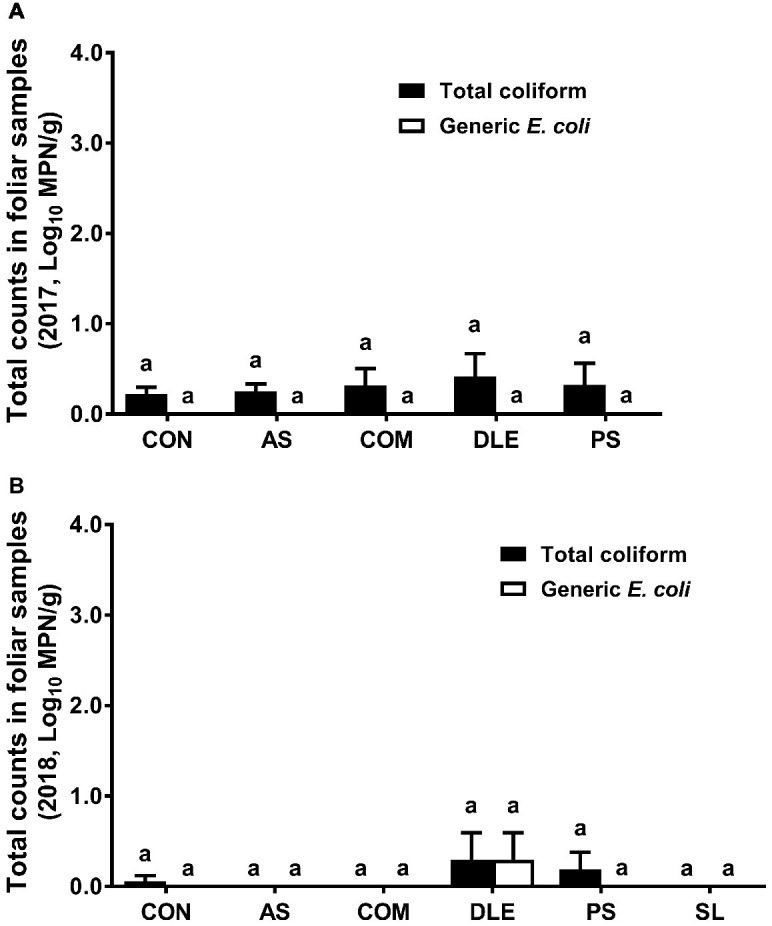
Most probable number of total coliform and generic *E. coli* in foliar samples from raspberry field in both 2017 and 2018 cropping seasons. **(A)** Total coliform and generic *E. coli* in 2017; **(B)** total coliform and generic *E. coli* in 2018. Mean ± SEM, 12 samples were collected per treatment in 2017 and eight samples per treatment in 2018, while 25 g per sample was analyzed. Histogram bars without common letter differ significantly (*p* < 0.05). CON: standard fertilization; AS: ammonium sulfate; COM: compost, DLE: digested liquid effluent; PS: phosphorous solid; SL: straight lagoon. MPN: most probable number.

For both cropping seasons, total coliform or generic *E. coli* counts in foliar samples were lower than 0.4 Log_10_ MPN/g regardless of fertilizer treatment (Figure 4). Neither total coliform nor generic *E. coli* was detected by the MPN method in raspberry fruit samples for 2 years (data not shown).

### Detection of Pathogenic Microorganisms in Fertilizer, Soil, Foliage, and Fruit Samples

In the 2-year study, no STEC or *L. monocytogenes* was detected from fertilizer, soil, foliar, or raspberry fruit samples throughout the sampling period (Data not shown). In 2017 cropping season, *Salmonella* was not detected in soil samples before fertilizer application as well as all soil samples post-fertilization except PS (Table 2). *Salmonella* in soil amended with PS was reduced to undetectable level 2 or 4 months post-application (Table 2). No *Salmonella* was detected in foliar or raspberry fruit samples (Table 2). In 2018, *Salmonella* was not detected in fertilizers except SL and DLE (Table 2). MPN enumeration showed that DLE fertilizer contained 55.5 ± 12.5 MPN/g of *Salmonella* while SL fertilizer contained less than detection limit (3 MPN/g) (Table 2). Consistently, *Salmonella* was detected in soil samples right after amendment with these two fertilizers (Table 2). *Salmonella* was also detected in soil samples pre-application of DLE or right after application of CON or AS (Table 2). *Salmonella* populations in all positive soil samples were less than 12 MPN/g (Table 2). In agreement with 2017, no *Salmonella* was detected in soil samples 2 and 4 months post-fertilizer application, foliar or fruit samples regardless of fertilizer treatments in 2018 (Table 2).

**Table 2:** Detection of *Salmonella* in raspberry field in 2017–2018 production seasons.^a^

Treatment	Fertilizer	Soil				Foliar	Fruit
		Mar-Apr		Jun	Aug		
		Pre	Post				
CON	0/5∣0/4^b^	0/8^c^	0/8∣**1/8** (5.4 ± 5.4)	0/8	0/8	0/12∣0/8	0/12
AS	0/5∣0/4	0/8	0/8∣**1/8** (2.0 ± 2.0)	0/8	0/8	0/12∣0/8	0/12
COM	0/5∣0/4	0/8	0/8	0/8	0/8	0/12∣0/8	0/12
DLE	0/5∣**4/4** (55.5 ± 12.5)	0/8∣**2/8** (< LOD)	0/8∣**2/8** (< LOD)	0/8	0/8	0/12∣0/8	0/12
PS	**5/5**∣0/4 (/)	0/8	**2/8**∣0/8 (/)	0/8	0/8	0/12∣0/8	0/12
SL	**1/4** (< LOD)	0/8	**1/8** (11.6 ± 11.6)	0/8	0/8	0/12∣0/8	0/12

a *Results were confirmed by Latex *Salmonella* and PCR detection of *invA* gene*;

b *Positive samples/total samples per treatment in 2017∣Positive samples/total samples per treatment in 2018 [enumeration of *Salmonella* in positive samples using most probable number (MPN), expressed in MPN/g, Mean ± SEM, LOD: limit of detection, 3 MPN/g; /: not enumerated]*.

c *2017 and 2018 production season with the same result was only reported once*.

*CON: standard fertilization; AS: ammonium sulfate; COM: compost; DLE: digested liquid effluent; PS: phosphorous solid; SL: straight lagoon; Pre: pre-application of fertilizer; Post: post-application of fertilizer. Jun: soil was sampled in June before fruit harvest; Aug: soil was sampled in August after fruit harvest*.

## Discussion

### Fecal Indicator Microorganisms in Raspberry Field

Raw diary manure (SL) in this study contained ~3.0 and 2.6 Log_10_ CFU/g of total coliform and generic *E. coli*, respectively. Similarly, harvested cattle manure is reported to carry 3.2 and 2.5 Log_10_ CFU/g of total coliform and generic *E. coli*, respectively (Klein et al., 2010). Raw bovine manure collected from another farm contained ~4.2 Log_10_ CFU/g of generic *E. coli* (Chiapetta et al., 2019). The US Food and Drug Administration (FDA) requires biological soil amendments of animal origin to contain less than 1,000 MPN (3 Log_10_ MPN) fecal coliforms per gram or milliliter of total solids after a valid controlled treatment process (FDA, 2018b). All the treated fertilizers tested in this study met the above standard. In support of our finding, *E. coli* in dairy manure was reduced to undetectable levels during composting process (Larney et al., 2003; Grewal et al., 2006). Both solid and liquid of plug-flow anaerobically digested bovine manure contained 1.6–2.5 Log_10_ CFU/g of generic *E. coli* (Chiapetta et al., 2019), while another study reported that anaerobically digested dairy manure contained 4.5–5.4 Log_10_ CFU/g of generic *E. coli* (Saunders et al., 2012).

Soil samples before fertilizer application in 2018 cropping season had ~3.4 Log_10_ CFU/g total coliform, which was lower than that in 2017, but similar with that of ~2.8 Log_10_ CFU/g in lettuce field pre-application of farmyard manure (Fischer-Arndt et al., 2010) and ~3.4 Log_10_ MPN/g in untreated soil from vegetable field (Machado et al., 2006). The total coliform level in soil samples was fairly stable across the 2017 cropping season regardless of fertilizer treatment, while it increased with time before harvesting fruit in 2018 and reached a level similar to that of the 2017 season. The exact reason for the observed pattern was uncertain, which might be due to the weather and other environmental factors such as temperature associated with different cropping seasons (Saunders et al., 2012). Generic *E. coli* in 2018 cropping season remained at less than 1.0 Log_10_ CFU/g in soil samples throughout the production season regardless of fertilizer treatments. Similarly, generic *E. coli* in soil post-application of raw or anaerobically digested dairy manure stayed at 1.0–2.0 Log_10_ CFU/g soil for at least 20 days (Saunders et al., 2012). In contrast to the stable generic *E. coli* level and the increased coliform counts in soil samples collected over the 2018 production season, *Salmonella* was only detected in soil samples collected pre- and post-fertilizer application. This is consistent with previous finding that generic *E. coli* is a poor fecal indicator in soil from fresh produce field (Ceuppens et al., 2015). Generic *E. coli* can be present in non-fecal environment and has the ability to proliferate in the environment (Park et al., 2016; Patterson et al., 2018; Allard et al., 2019). The above evidence collectively reinforces the need to explore a suitable generic fecal indicator microorganism of soil.

### Pathogenic Microorganisms in Raspberry Field

#### Shiga Toxin-Producing *E. coli*

STEC are frequently involved in deadly fresh produce outbreaks (CDC, 2014, 2018a,b). Both traditional culture-based method and real-time PCR method were carried out to detect STEC from the collected samples during 2017 and 2018 cropping seasons. Traditional culture method takes more than 24 h to generate results while PCR provides fast and real-time screening. However, naturally occurring PCR inhibitors in environmental samples can impair direct DNA amplification by PCR and lead to false-negative results (Dobhal et al., 2014). Two methods were carried out simultaneously as a comparison. No STEC was detected from the tested fertilizers, indicating the treatments of dairy manure are adequate to eliminate the potential STEC in the respective raw manure. Similarly, *E. coli* O157:H7 was not detected in the lettuce grown in soil amended with bovine manure-derived fertilizers including firm manure and slurry (Johannessen et al., 2004).

#### Salmonella

*Salmonella* were detected in PS (2017), DLE (2018), and SL (2018) treatments and further transferred to the amended soil samples. In support of our finding, 6.6% of solid bovine manure collected from dairy farms in California was positive of *Salmonella* (Chen et al., 2019). *Salmonella* has been detected in more than 80% of effluent bovine manure immediately post-commercial anaerobic digestion (Chiapetta et al., 2019). The *Salmonella* in raw manure (SL) was below our detection limit of 3 MPN/g while DLE was 55.5 ± 12.5 MPN/g, higher than the microbial standard of 3 MPN/4 g for treated manure (FDA, 2018b). A recent study reported that *Salmonella* in raw dairy manure ranged from less than 3 Log_10_ CFU/g to 5.2 Log_10_ CFU/g (Chen et al., 2019). Farm-scale psychrophilic anaerobic digester only reduced *Salmonella* by 1.2–1.4 Log_10_ CFU/g in swine manure (initial *Salmonella* population ranged from less than 2.0 to 4.7 Log_10_ CFU/g), resulting in fair amount of *Salmonella* remaining in the effluent manure (Masse et al., 2011). In contrast, thermophilic and mesophilic anaerobic digesters reduced *Salmonella* in raw cattle manure from 1,000–1,500 MPN/g to less than 1 MPN/g (Harikishan and Sung, 2003).

*Salmonella* was also detected in two of the soil samples pre-application of DLE and one of the soil samples post-application of CON and AS treatments, which were free from *Salmonella*, in the 2018 cropping season. It has been reported that 2.6% of 617 soil/sediment samples collected from a major produce region in California was positive of *Salmonella* (Gorski et al., 2011). Another study also showed that *Salmonella* was detected in 2.2% of 178 soil samples collected from five produce farms in New York State (Strawn et al., 2013). The uneven distribution of *Salmonella* in soil might contribute to our observation. It could also be due to the transmission through aerosol in high-wind condition, wild animals, or stormwater runoff (Baertsch et al., 2007; Silva et al., 2014). *Salmonella* was below detectable level in soil samples 2 and 4 months post-fertilizer application, foliar, or raspberry fruit samples in both cropping seasons. In support of our finding, *Salmonella* contaminated in soil at ~3.0 Log_10_ CFU/g survived for less than 2 months (Nicholson et al., 2005). Lettuce grew in soil contaminated with ~4.0 Log_10_ CFU/g. *S.* Typhimurium was negative of *Salmonella* even though *Salmonella* was still detectable in the soil samples at lettuce sampling (Franz et al., 2005). However, *S.* Typhimurium at an initial contamination level of ~7.0 Log_10_ CFU/g persisted in soil for more than 5 months, and was detected in lettuce and parsley that grew in this contaminated soil (Islam et al., 2004b).

#### L. monocytogenes

In this study, no *L. monocytogenes* was detected from any of the collected samples in both cropping seasons. Our result indicated that the current dairy manure treatments adequately minimized the potential risk of introducing *L. monocytogenes* to raspberry production system. In support of our finding, *L. monocytogenes* was not detected from treated bovine or human waste, soil, or vegetables (tomato, radish, carrot, cucumber, pepper, and lettuce) in field trials (Johannessen et al., 2004; Brochier et al., 2012; Rahube et al., 2014).

## Conclusion

Based on 2 years of field studies conducted in 2017 and 2018 raspberry cropping system, application of raw dairy manure straight lagoon (SL), anaerobically digested dairy manure liquid effluent (DLE), aerobically composted dairy manure (COM), and refined anaerobically digested dairy manure products including ammonium sulfate (AS) and phosphorous solid (PS) had no major impacts on indicator microorganisms including total coliform and generic *E. coli* as well as the major foodborne pathogens including STEC, *Salmonella,* and *L. monocytogenes* in red raspberry production system in Lynden sandy loam. Application of raw dairy manure or its derivatives once per season 4 months pre-harvest did not introduce microbiological safety risk to red raspberry production under good agricultural practices.

## Data Availability Statement

All datasets generated for this study are included in the manuscript/supplementary files.

## Author Contributions

LS, XS, YS, and H-CT performed the microbial experiment. LS wrote the manuscript. CB and ES collected samples for microbial analyses. M-JZ, LS, CK, and CB designed the study. M-JZ, CK, CB, and MD revised the manuscript.

### Conflict of Interest

The authors declare that the research was conducted in the absence of any commercial or financial relationships that could be construed as a potential conflict of interest.
